# Comparison of phenotypic methods for the detection of carbapenem non-susceptible *Enterobacteriaceae*

**DOI:** 10.1186/1757-4749-6-13

**Published:** 2014-05-19

**Authors:** Andrea Bartolini, Ilaria Frasson, Antonietta Cavallaro, Sara N Richter, Giorgio Palù

**Affiliations:** 1 Padua Hospital, Microbiology and Virology Unit, via Giustiniani 2, Padua 35121, Italy; 2 Department of Molecular Medicine, University of Padua, via Gabelli 63, Padua 35121, Italy

**Keywords:** Carbapenemases, Phenotypic detection, Genotypic assay, Molecular analysis, *Enterobacteriaceae*

## Abstract

**Background:**

Multidrug resistance and, in particular, carbapenem resistance is spreading worldwide at an alarming rate, comprehending a variety of bacterial species and causing both nosocomial and community acquired outbursts. Early and efficient detection of infected patients or colonized carriers are mandatory steps in infection control and prevention of multidrug resistance diffusion. The latest EUCAST guidelines for detection of carbapenemase-producing *Enterobacteriaceae* have set low clinical breakpoints to ensure the maximum detection sensitivity of positive samples. Current workflows involve an initial screening step for species and resistance pattern detection, followed by phenotypic and/or genotypic confirmation. The aim of the present study was to assess the efficiency of six widely used and validated phenotypic assays for the detection of carbapenemases/AmpC in *Enterobacteriaceae*, to estimate the best workflow in the routine characterization of *Enterobacteriaceae* isolates.

**Methods:**

A panel of 108 non-repetitive *Enterobacteriaceae* isolates with reduced susceptibility to carbapenems was analyzed by means of 1) Modified Hodge Test, 2) Metallo Beta Lactamase Etest, 3) Double disk test with EDTA, 4) Rosco Diagnostica KPC and MBL confirm kit (RDCK™), 5) AmpC Etest and 6) Cloxacillin inhibition test. Confirmation and validation of results was achieved by genotypic analysis.

**Results:**

The most accurate identification of resistance determinants was obtained with the combined disc test (Rosco Diagnostica KPC and MBL confirm kit) which had to be coupled with the cloxacillin inhibition test for correct detection of AmpC enzymes. However, in general, phenotypic tests failed to characterize isolates harboring multiple carbapenem resistance determinants, which were successfully assessed only by PCR-based analysis.

**Conclusions:**

To detect and control the spread of pathogens with complicated resistance patterns, both optimized phenotypic analysis (i.e. Rosco Diagnostica KPC and MBL confirm kit coupled with the cloxacillin inhibition test) and genotypic assays are recommended in the routine diagnostic of clinical laboratories.

## Background

Multidrug resistance among *Enterobacteriaceae* has become a worldwide major public health issue. The most worrisome emerging resistance feature corresponds to the production of carbapenem-hydrolysing beta-lactamases [1]. Carbapenems are considered the last line of effective therapy available for the treatment of severe infections [2,3]; resistance to these agents reduces clinical therapeutic choices and frequently leads to treatment failure. Carbapenemase enzymes include class A carbapenemases (KPC types), class B or metallo-beta-lactamases (MBLs) (VIM, IPM, and NDM types), and class D oxacillinases (e.g., OXA-48-like enzymes). In addition, decreased susceptibility to carbapenems in *Enterobacteriaceae* may be caused by either extended spectrum beta-lactamases (ESBLs) or AmpC enzymes combined with drug decreased permeability, due to loss of porins [4]. The association of multiple resistance determinants, comprehending carbapenemase, cephalosporinase enzymes and ESBLs, poses a challenge in the routine microbiologic diagnostic of *Enterobacteriaceae*[4,5]. Different phenotypic and molecular screening and confirmatory tests have been described [6-8]. Currently, the resistance detection workflow involves an initial screening step followed by phenotypic and/or genotypic confirmation [8-10]. The screening step is based on evaluation of strain susceptibility to carbapenems, measured as MIC and compared to MIC values of ATCC control strains. Current EUCAST clinical breakpoints for the detection of carbapenemase-producing *Enterobacteriaceae* are set to 0.5 mg/L for ertapenem and 2 g/ml for meropenem and imipenem, while the screening cutoff are set to 0.12 mg/L for meropenem and ertapenem and 1 g/L for imipenem [9,10]. This choice reflects the presence of carbapenemase producers that poorly express resistance determinants and would thus be categorized as susceptible by clinical breakpoints, while they need further screening for correct identification.

The phenotypic confirmation of carbapenemase production is based on the detection of diffusible carbapenemases (evaluated in the Modified Hodge Test (MHT)) or on inhibition of carbapenemase activity, detected in phenotypic assays based on the synergy between MBL or KPC inhibitors and carbapenems [11,12]. The most widely used inhibitors are the metal-chelating agent EDTA and dipicolinic acid against MBL, boronic acid for Ambler class A carbapenemases and cloxacillin against AmpC. These are used in different formats which include the double-disk synergy test, the combined disk test, and carbapenem/carbapenem-EDTA or cefotetan/cefotetan-cloxacillin Etest strips [13-15]. In contrast, the genotypic confirmation is based on PCR-based techniques.

In this work we aimed at establishing the best workflow based on phenotypic analysis in the routine detection and characterization of carbapenem resistant *Enterobacteriaceae* isolates. We compared the efficiency of six different phenotypic assays: 1) MHT, 2) MBL Etest, 3) Double disk test with EDTA, 4) Rosco Diagnostica KPC and MBL confirm kit (RDCK™), 5) AmpC Etest and 6) Cloxacillin inhibition test in detecting resistance determinants on a panel of *Enterobacteriaceae* isolates with reduced susceptibility to carbapenems. Results were validated by comparison with genotypic data. The RDCK™ was the most reliable assay in our hands; however, all phenotypic tests failed to detect multiple carbapenem resistance mechanisms, which needed to be assessed by genotypic analysis. Therefore, the optimized workflow to diagnose and control the spread of pathogens with complicated resistance patterns must involve both genotypic and phenotypic analysis.

## Results

### Antimicrobial susceptibility and molecular characterization of bacterial isolates

One hundred and eight non-repetitive enterobacterial clinical isolates that showed reduced susceptibility to carbapenems with MIC values ≥ 0.5 mg/L of imipenem, meropenem or ertapenem, at the Vitek II analysis, were chosen for the study. The challenge set of isolates included 93 *Klebsiella pneumoniae*, 2 *Klebsiella oxytoca*, 7 *Enterobacter cloacae*, 2 *Enterobacter aerogenes*, and 4 *Escherichia coli*.

Molecular characterization was performed on the complete set of isolates. PCR and real time PCR reactions were performed as previously described [16-18]. The resistance determinants, i.e. *bla*_KPC_, *bla*_VIM_, *bla*_OXA-48_, *bla*_NDM_, plasmidic AmpC genes and *bla*_CTX-M1-type_, were amplified and sequenced. Genotypic results indicated that the isolates included 87 KPC-, 5 VIM-, 3 KPC- and VIM-, 1 NDM-1-, 5 OXA-48-, 4 AmpC- and 3 ESBL-positive samples (Table 1). ESBL-positive sample harbored CTX-M-15. MICs of carbapenems of the KPC-, VIM-, OXA-48- and NDM-producers were ≥ 0.5 mg/L. In contrast, the AmpC-positive samples showed a reduced susceptibility to imipenem and meropenem, with MICs ≥ 0.25 mg/L, and slight resistance to ertapenem with MICs ≥ 4 mg/L. These data confirmed the necessity to further test isolates with reduced susceptibility, as indicated in the EUCAST guidelines (Table 1) [9,10]. Moreover, 5 negative control strains, one per species, were included: they all tested negative for carbapenem resistance determinants in the molecular assays.

**Table 1:** Resistance phenotypes of the tested enterobacterial isolates and negative controls, expressed as MIC ranges of carbapenems obtained at the Vitek II

**Isolates #**	**Species**	**Resistance determinants**	**Ertapenem MIC range (mg/L)**	**Imipenem MIC range (mg/L)**	**Meropenem MIC range (mg/L)**
84	*K. pneumoniae*	KPC	4 - > 8	4 - > 16	2 - > 16
2	*E. coli*	KPC	≤ 0,5 - 4	8	1
1	*E. cloacae*	KPC	> 8	> 16	> 16
3	*K. pneumoniae*	KPC + VIM	> 8	> 16	> 16
3	*E. cloacae*	VIM	> 8	> 16	> 16
2	*K. oxytoca*	VIM	≤ 0,5	1- > 16	1- > 16
1	*K. pneumoniae*	NDM	> 8	> 16	> 16
3	*K. pneumoniae*	OXA-48	4 - > 8	2 - > 16	1 - 2
1	*E. coli*	OXA-48	1	2	≤ 0,25
1	*E. cloacae*	OXA-48	2	1	≤ 0,25
2	*E. aerogenes*	AmpC	> 8	> 16	8 - > 16
2	*E. cloacae*	AmpC	4	≤ 0,25 - 1	≤ 0,25
2	*K. pneumoniae*	ESBL	> 8	1	4
1	*E. coli*	ESBL	> 8	> 16	> 16
**Controls**					
NC1	*E. coli*	none	≤ 0,5	≤ 0,25	≤ 0,25
NC2	*E. cloacae*	none	≤ 0,5	0,5	≤ 0,25
NC3	*E. aerogenes*	none	≤ 0,5	2	≤ 0,25
NC4	*K. pneumoniae*	none	≤ 0,5	≤ 0,25	≤ 0,25
NC5	*K. oxytoca*	none	≤ 0,5	0,5	≤ 0,25

Enterobacterial samples were grouped bases on the species and resistance determinant/s.

NC: negative control.

### Sensitivity and specificity of selected commercial phenotypic tests

The panel of genotypically characterized *Enterobacteriaceae* with reduced susceptibility to carbapenems was next assessed with six different phenotypic detection methods: 1) Modified Hodge test (MHT), 2) MBL Etest, 3) EDTA-double disk test, 4) Rosco Diagnostica KPC and MBL confirm kit (RDCK™) 5) AmpC Etest and 6) cloxacillin-inhibition test. Tests 5) and 6) are normally used the verify the presence of AmpC in samples displaying lower susceptibility to carbapenems but testing negative in the carbapenemase specific assays 1)-4). Results are summarized in Table 2.

**Table 2:** **Comparison of phenotypic and genotypic analysis methods for the detection of carbapenem-resistance determinants in a panel of 108 carbepenem non-susceptible *Enterobacteriaceae***

		**MHT**		**Etest MBL**		**EDTA DDST**		**RDCK™**		**Molecular tests**	
**RD**	**N.**	**ID**	**ND**	**ID**	**ND**	**ID**	**ND**	**ID**	**ND**	**ID**	**ND**
KPC-2/3	87	87	0	0	87	0	87	87	0	87	0
VIM-1/2	5	4	1	5	0	5	0	3	2	5	0
KPC-2 plus VIM-1	3	2	1	0	3	0	3	0	3^*^	3	0
NDM-1	1	0	1	1		1	0	1	0	1	0
OXA-48	5	2	3	0	5	0	5	5	0	5	0
Total	101	95/101		6/11		6/11		96/101		101/101	
Technique sensitivity/specificity		Sensitivity 94%		Sensitivity 54.5%		Sensitivity 54.5%		Sensitivity 95%		Sensitivity 100%	
		Specificity 100%		Specificity 100%		Specificity 100%		Specificity 99%		Specificity 100%	

MTH: Modified Hodge Test; DDST: Double Disk Synergy Test; RDCK ™: Rosco Diagnostics Confirmation Kit; RD: resistance determinants; N.: number of isolates; ID: identified; ND: not detected; ^*^in one case the isolate harboring KPC and VIM was misclassified as OXA-48 producer, in two other cases the results were doubtful.

AmpC- and ESBL-positive strains were not detected by MHT, Etest MBL and EDTA DDST while molecular tests correctly identified both AmpC and ESBL determinants.

RDCK™ did not indicated the presence of ESBL determinants as indicated by manufacturer’s instructions and misclassified as OXA-48 producer 2 out of 4 AmpC producers.

1) MHT correctly identified 95/101 carbapenemase producers (94% sensitivity, 100% specificity). KPC-positive isolates were all successfully identified (87/87, 100% sensitivity, 100% specificity), while sensitivity lowered when testing VIM-positive and KPC-/VIM-positive samples. Less than half OXA-48-positive samples were recognized by MHT, while the NDM-1 positive sample gave a disturbed edge of the inhibition zone that did not lead to straightforward identification of the positive sample. As for the AmpC-positive *Enterobacter spp* strains, 3/4 samples yielded growth patterns of doubt interpretation that could be erroneously classified as positive. Finally, MHT correctly reported no positive samples in ESBL-producing strains, despite their upraised carbapenem MICs, in line with reports by other authors [19].

2) and 3) Both the MBL Etest and the double disk test recognized 6/6 VIM- and NDM-positive samples. However, both assays completely failed to identify the presence of MBL in isolates harboring both VIM and KPC, despite the high MICs of carbapenems of these samples. The inefficacy of synergy tests using a single inhibitor for the characterization of isolates expressing both Ambler class A and B has been previously reported [20]. Furthermore, the MBL Etest and EDTA double disk synergy test were tested against the whole panel of isolates and no false positive result was recorded.

4) RDCK™ correctly pointed out the presence of carbapenem-hydrolyzing enzymes in 96/101 isolates. In detail, the commercial phenotypic test indicated the presence of KPC, NDM-1 and OXA-48, in 93/93 samples (100% sensitivity, 100% specificity). The assay identified 3/5 VIM-positive samples, while failed in pointing out the double positive isolates that harbored both KPC and VIM. Out of the 4 *Enterobacter spp.* samples, two *E. cloacae* and two *E. aerogenes*, RDCK™ revealed the presence of AmpC expression in the two *E. aerogenes* samples. The two AmpC-positive *E. cloacae* samples tested either negative or suggested the presence of an OXA-48 like enzyme. Conversely all *Enterobacter spp.* samples were found positive for plasmidic AmpC by PCR analysis. This fact highlights the possibility to obtain false positive or misleading results with the RDCK™ kit. The same outcome was recently reported by other groups [7,21]. The kit did not recognize the presence of ESBL overexpression with porin impairment, which is in accordance with the test guidelines.

5) and 6) The phenotypic identification of AmpC enzyme conferring carbapenem resistance was conducted by means of AmpC Etest and cloxacillin-containing medium. AmpC Etest correctly identified only 2/4 samples, while cloxacillin inhibition test succeeded in identifying 4/4 AmpC-positive isolates.

## Discussion and conclusions

The massive worldwide spreading of carbapenemase-producers, mainly *Enterobacteriaceae*, has forced routine analysis to elaborate reliable detection methods. High sensitivity and specificity together with a rapid workflow has become mandatory to delineate the treatability of dangerous pathogens and to control and hinder their spread. The epidemiology of carbapenemases had been widely discussed as it has become a major health issue, especially in countries, such as Greece, Israel, USA and many others, where carbapenemase producers are becoming endemic [1]. A large variety of carbapenem-hydrolyzing enzymes has been identified in Gram negative bacilli [22]. The Ambler class A (KPC-type) and class B (VIM- and NDM-type) are the most relevant carbapenemases in the clinic; class D (OXA-48-like) are gaining increasing importance, due to their recent spread and to their peculiar hydrolysis profile [23]. Moreover a particular class of plasmidic cephalosporinases (AmpC, Ambler class C) displays a slightly extended inducible hydrolytic activity towards carbapenems and therefore has to be taken into consideration during putative carbapenemase detection and subsequent antimicrobial treatment [24]. In the present work we analyzed six different phenotypic tests for their ability to correctly identify carbapenem resistance mechanisms, to provide the most accurate, reliable and easy to set up workflow for the detection of carbapenemase and carbapenem hydrolyzing AmpC producers in clinical specimens.

The threshold of susceptibility to the three major carbapenems (imipenem, meropenem or ertapenem) was set to 0.5 mg/L: considering the latest EUCAST document on “Detection of resistance mechanisms” [9,10], the chosen screening cutoff offers a broad and safe probability of detection of carbapenemase producers. A similar threshold has been set by other groups (MIC of meropenem ≥ 0.5 mg/L as point of suspicion of carbapenemase production [20]). Although a low threshold leads to inclusion in routine diagnostic of isolates that may result negative in subsequent characterization of carbapenem-resistance, this choice maximizes detection sensitivity and should be recommended for epidemiology and spreading control purposes. In addition, our results suggest to take into consideration the MIC of the three main carbapenems used in the clinical practice, i.e. imipenem, meropenem and ertapenem, as each of them displays both positive and negative features: reduced screening breakpoints to imipenem and meropenem increase sensitivity but decrease specificity of carbapenemase detection, as the MIC distribution of the wild-type population is very variable and may be several times higher than the breakpoint. Meropenem offers the best compromise between sensitivity and specificity in terms of detection of carbapenemase-producers. Ertapenem exhibits low efficiency in indicating the presence of non-KPC class A carbapenemases and in general low specificity for carbapenemase detection, since AmpC/ESBL positive Enterobacter spp isolates, have higher MICs of ertapenem than of imipenem and meropenem [13,25]. To avoid resistance spreading, we would not recommend setting threshold or screening cutoff higher than those indicated by EUCAST.

Among the tested phenotypic assays, we found important differences in terms of sensitivity and specificity. To summarize, the MHT (1) worked well for the detection of KPC, while it was not able to consistently recognize MBLs. In addition, it has been reported that high levels of expression of AmpC coupled with decreased permeability may be interpreted as carbapenem hydrolyzing enzyme and therefore may yield false positive results [19,21]. The MHT has been the gold standard technique in the past years [26]; however, the massive spreading of both MBL and OXA-48-like enzymes, coupled with the diffusion of Ambler class A carbapenemases in Gram negative bacilli, such as *Proteus spp.* and *Pseudomonas spp.*, makes it less reliable nowadays [27]. The successful detection of MBLs was mainly achieved by the EDTA inhibition test, using indifferently Etest strips (2) or the double disk test (3). The main limit of these assays is that they may fail to detect positive isolates with low level resistance [26]. Moreover, in both assays only KPC was detected in samples positive for both KPC and MBL. To our experience, the strips are less sensitive than the double disk test; on the other hand, EDTA containing discs are manually prepared which increases the statistical error. The RDCK™ (4) was the best choice for the phenotypic detection of carbapenemase producers which expressed enzymes belonging to Ambler class A, B, C and D. The latest EUCAST guidelines recommend the use of this kit in routine diagnostic [9,10]. Results were achieved within 24 hours from the first identification of diminished susceptibility to carbapenems at the Vitek II. However, we found interpretation of results disputable in the case of OXA-48-like enzymes and AmpCs, which thus needed to be confirmed by other techniques. Results obtained by this method may be improved introducing temocillin or combining two different inhibitors, such as boronic acid and dipicolinic acid [7,20].

Other groups have also recommended the improvement of RDCK™ in terms of sensitivity of the meropenem/DPA combination, in particular for the detection of IMP enzymes (IMP-8), and the evaluation of local epidemiology prior to utilization of this phenotypic test in the routine diagnostic [28,29]. The identification of overexpressed AmpC enzymes, which display a broadened hydrolysis spectrum conferring carbapenem resistance [30], resulted sensitive and specific using the cloxacillin-containing medium (6), as previously reported [13]. In contrast, the Etest strips (5) lacked sensitivity as they failed to detect 50% of positive isolates. To note that none of the tested phenotypic assays was able to detect isolates positive for two resistance determinants, i.e. KPC and VIM. This problem has to be taken into serious consideration, since bacilli with multiple carbapenem resistant mechanisms are increasingly encountered. Only two studies have so far addressed this issue reporting that clinical isolates expressing more than one carbapenemase in association with other beta-lactamases, such as ESBLs, display composite phenotypic resistance profiles that available phenotypic assays are unable to correctly dissect [7,26]; the EUCAST guidelines do not thoroughly cover this aspect yet.

On the whole, phenotypic analysis was less reliable than genotypic characterization in the identification of carbapenem resistant strains. However, PCR-based molecular assays have their own limitations: they need expensive equipment and reagents, and expert personnel which are not always available to the diagnostic laboratory. In addition, primers have to be designed within low mutation rate regions and the use of specific primers hinders the identification of novel resistance genes, possibly reporting false negative results.

Therefore, among phenotypic methods for the detection of carbapenemase producers, in our hands RDCK™ reported the most reliable results. This method, as indicated in the EUCAST guidelines, should be applied in the routine screening of all samples with reduced susceptibility to carbapenems. In case of negative results in carbapenem non-susceptible strains, further analysis by means of cloxacillin inhibition test is suggested for the unambiguous detection of AmpC enzymes. In addition, if RDCK™ gives doubtful results, the simultaneous presence of two or more carbapenem resistance mechanisms, or an OXA-48-like enzyme, should be suspected and the molecular tests are the methods of choice in this instance. Therefore, where possible, the concomitant use of both phenotypic and genotypic analysis is highly recommended. Molecular tests can be applied straightforward to screen putative infected/colonized patients/carriers in intensive care or transplant units and immunocompromised patients. Quickness to achieve results, obtained by PCR-based methods, is crucial in terms of clinical management, implementation of infection control measures and for antibiotic stewardship [31]. In conclusion, to our experience, the best workflow applicable in routine phenotypic diagnostics of carbapenem non-susceptible *Enterobacteriaceae* involves the application of the updated EUCAST clinical breakpoints and the use of RDCK™ as first line analysis, followed by, in case of doubtful results, AmpC (i.e. cloxacillin containing medium) and OXA-48 (i.e. evaluation of temocillin resistance) confirmation tests. Molecular assays are also recommended due to their rapidity and sensitivity in the characterization of isolates displaying a complex phenotype, whose identification of resistance mechanisms would be time-consuming and challenging at the phenotypic level.

## Materials and methods

### Bacterial strains

One hundred and eight, non-repeat clinical isolates of carbapenemase-, AmpC- and ESBLs producing *Enterobacteriaceae* were included in the study. They were collected in the Microbiology and Virology Unit of the Padua Teaching Hospital from both hospitalized and non-hospitalized patients. These encompassed *Klebsiella spp*., *Escherichia coli*, and *Enterobacter spp*.. All these isolates tested non-susceptible (i.e., intermediate or resistant) to carbapenems (i.e., MICs of meropenem, imipenem and ertapenem were at least 0.5 mg/L) as determined by the Vitek II system (bioMerieux, Marcy l’Etoile, France). Moreover, the disk diffusion test was performed to detect ESBLs (BD BB™ Sensi-Disk™, Becton Dickinson Italia, Milan, Italy). Five additional carbapenemase-negative control strains (ATCC strains number 25922 for *E. coli*, 700603 for *K. pneumoniae*, 700324 for *K. oxytoca* and two clinical samples for *E. cloacae* and E. *aerogenes*, assessed by molecular analysis) were also included as negative controls.

### Molecular analysis

PCR and real-time PCR amplification were performed as previously described [16-18]. Bacterial lysates were obtained by heat shock from single colonies picked up from purity plates. DNA was also extracted from stools, when necessary. Subsequent purity plates were analysed by means of traditional PCR and gene sequencing for further confirmation. The presence of *bla*_KPC_, *bla*_VIM_, *bla*_OXA-48_, *bla*_NDM_, plasmidic AmpC genes and *bla*_CTX-M1-type_ was investigated [32,33]. PCR amplicons were sequenced by end-primers in an ABI3130xl sequencer (Applied Biosystems, Life Technologies Italia, Milan, Italy) and the obtained sequences were compared to the corresponding sequences in the NCBI database.

### Phenotypic tests

#### Modified Hodge test

Carbapenemase activity assay was performed as previously reported [26]. In brief, *E. coli* ATCC 25922 was streaked for confluent growth on cation-adjusted Mueller-Hinton II agar plates (Becton Dickinson Italia, Milan, Italy). A disk saturated with 10 μg of imipenem (Becton Dickinson Italia, Milan, Italy) was placed in the center of the plate, and each sample was subsequently streaked from the disk to the edge of the plate. The presence of a distorted inhibition zone after overnight incubation was interpreted as a positive result. Performance quality control strains were included in each test (the positive control was *K. pneumoniae* ATCC BAA-1705, the negative control was *K. pneumonia*e ATCC BAA-1706).

#### EDTA inhibition test

This combination phenotypic detection method was performed using meropenem and meropenem-EDTA (292 μg). The stock solution of EDTA was prepared by dissolving anhydrous EDTA (Sigma-Aldrich, Milan, Italy) in distilled water at a concentration of 0.1 M. 10 μL of this solution were dispensed onto meropenem discs (BD BB™ Sensi-Disk™, Becton Dickinson Italia, Milan, Italy). The discs were then dried and used within 60 min. The test was performed as a standard diffusion method, using one disk of meropenem and one containing meropenem and EDTA. A growth zone difference above 5 mm confirmed the presence of MBL [34].

#### Rosco Diagnostica KPC and MBL confirm kit (RDCK™)

This commercial Kit (catalog number 98006) consists of 4 tablets: tablet A contains meropenem, tablet B contains meropenem and dipicolinic acid (MBL inhibitor), tablet C contains meropenem and cloxacillin (AmpC inhibitor), and tablet D contains meropenem and boronic acid (KPC inhibitor). The results are interpreted as follows: the zone of inhibition of tablet A is compared to the zones of inhibition of each of the carbapenem-plus-inhibitor tablets (B, C, and D). A growth zone difference above 5 mm indicates the presence of enzyme activity. Each tablet indicates one specific resistance mechanism: tablet B points to MBL activity; tablet D reveals KPC activity, the association of tablets C and D shows AmpC activity coupled with porin loss.

#### Cloxacillin inhibition test

Both cation adjusted and cloxacillin-containing (250 mg/ml) Mueller-Hinton II agar plates (Becton Dickinson Italia, Milan, Italy) were inoculated with isolate suspension, as described [13]. One cefotaxime (30 μg, BD BB™ Sensi-Disk™) and one ceftazidime (30 μg, BD BB™ Sensi-Disk™) disk were placed on both cation adjusted and cloxacillin-containing agar plates. After overnight incubation, an inhibition zone above 5 mm in the cloxacillin-containing plate revealed the presence of AmpC enzymes.

E-test MBL and AmpC: E-tests for detection of MBL and cefalosporinases AmpC were conducted according to manufacturer protocol (Etest® MP/MPI and CN/CNI, bioMerieux, Marcy l’Etoile, France).

## Abbreviations

EUCAST: The European Committee on Antimicrobial Susceptibility Testing; KPC: Klebsiella pneumoniae carbapenemase; VIM: Verona integron-encoded metallo beta lactamase; NDM: New Delhi metallo beta lactamase; MBL: Metallo beta lactamase; MIC: Minimum inhibitory concentration; ESBL: Extended spectrum beta lactamase; MHT: Modified Hodge test; RDCK™: Rosco Diagnostica confirmation kit; MP/MPI: Meropenem/meropenem plus inhibitor (EDTA); CN/CNI: Cefotetan/cefotetan plus inhibitor (cloxacillin).

## Competing interests

The authors declare that they have no competing interests.

## Authors’ contributions

AB carried out the phenotypic assays; IF carried out the genotypic analysis and drafted the manuscript; AB and IF analyzed the collected data; AC conceived of the study and participated in its design and coordination; SNR participated in study design and coordination and drafted/revised the manuscript; GP participated in the study coordination. All authors read and approved the final manuscript.

## References

[B1] Nordmann P Carbapenemase-producing Enterobacteriaceae: overview of a major public health challenge Med Mal Infect 2014 44 51 56 10.1016/j.medmal.2013.11.007 24360201

[B2] Munoz-Price LS Poirel L Bonomo RA Schwaber MJ Daikos GL Cormican M Cornaglia G Garau J Gniadkowski M Hayden MK Kumarasamy K Livermore DM Maya JJ Nordmann P Patel JB Paterson DL Pitout J Villegas MV Wang H Woodford N Quinn JP Clinical epidemiology of the global expansion of Klebsiella pneumoniae carbapenemases Lancet Infect Dis 2013 13 785 796 10.1016/S1473-3099(13)70190-7 23969216 PMC4673667

[B3] McLaughlin M Advincula MR Malczynski M Qi C Bolon M Scheetz MH Correlations of antibiotic use and carbapenem resistance in enterobacteriaceae Antimicrob Agents Chemother 2013 57 5131 5133 10.1128/AAC.00607-13 23836188 PMC3811461

[B4] Glasner C Albiger B Buist G Tambic Andrasevic A Canton R Carmeli Y Friedrich AW Giske CG Glupczynski Y Gniadkowski M Livermore DM Nordmann P Poirel L Rossolini GM Seifert H Vatopoulos A Walsh T Woodford N Donker T Monnet DL Grundmann H European Survey on Carbapenemase-Producing Enterobacteriaceae (EuSCAPE) Working Group Carbapenemase-producing Enterobacteriaceae in Europe: a survey among national experts from 39 countries, February 2013 Euro Surveill 2013 18 10.2807/1560-7917.es2013.18.28.20525 23870096

[B5] Canton R Ruiz-Garbajosa P Co-resistance: an opportunity for the bacteria and resistance genes Curr Opin Pharmacol 2011 11 477 485 10.1016/j.coph.2011.07.007 21840259

[B6] Dortet L Brechard L Cuzon G Poirel L Nordmann P Strategy for a rapid detection of carbapenemase-producing Enterobacteriaceae Antimicrob Agents Chemother 2014 58 2441 2445 10.1128/AAC.01239-13 24468779 PMC4023735

[B7] Miriagou V Tzelepi E Kotsakis SD Daikos GL Bou Casals J Tzouvelekis LS Combined disc methods for the detection of KPC- and/or VIM-positive Klebsiella pneumoniae: improving reliability for the double carbapenemase producers Clin Microbiol Infect 2013 19 E412 E415 10.1111/1469-0691.12238 23627340

[B8] Nordmann P Poirel L Strategies for identification of carbapenemase-producing Enterobacteriaceae J Antimicrob Chemother 2013 68 487 489 10.1093/jac/dks426 23104494

[B9] Breakpoint tables for interpretation of MICs and zone diameters http://www.eucast.org

[B10] Disk diffusion manual http://www.eucast.org

[B11] Giske CG Gezelius L Samuelsen O Warner M Sundsfjord A Woodford N A sensitive and specific phenotypic assay for detection of metallo-beta-lactamases and KPC in Klebsiella pneumoniae with the use of meropenem disks supplemented with aminophenylboronic acid, dipicolinic acid and cloxacillin Clin Microbiol Infect 2011 17 552 556 10.1111/j.1469-0691.2010.03294.x 20597925

[B12] Seah C Low DE Patel SN Melano RG Comparative evaluation of a chromogenic agar medium, the modified Hodge test, and a battery of meropenem-inhibitor discs for detection of carbapenemase activity in Enterobacteriaceae J Clin Microbiol 2011 49 1965 1969 10.1128/JCM.00203-11 21430097 PMC3122667

[B13] Willems E Verhaegen J Magerman K Nys S Cartuyvels R Towards a phenotypic screening strategy for emerging beta-lactamases in Gram-negative bacilli Int J Antimicrob Agents 2013 41 99 109 10.1016/j.ijantimicag.2012.07.006 23280443

[B14] Girlich D Halimi D Zambardi G Nordmann P Evaluation of Etest(R) strips for detection of KPC and metallo-carbapenemases in Enterobacteriaceae Diagn Microbiol Infect Dis 2013 77 200 201 10.1016/j.diagmicrobio.2013.08.002 24041554

[B15] Voulgari E Poulou A Koumaki V Tsakris A Carbapenemase-producing Enterobacteriaceae: now that the storm is finally here, how will timely detection help us fight back? Future Microbiol 2013 8 27 39 10.2217/fmb.12.130 23252491

[B16] Frasson I Biasolo MA Bartolini A Cavallaro A Richter SN Palu G Rapid detection of blaVIM-1-37 and blaKPC1/2-12 alleles from clinical samples by multiplex PCR-based assays Int J Antimicrob Agents 2013 42 68 71 10.1016/j.ijantimicag.2013.03.006 23642765

[B17] Richter SN Frasson I Biasolo MA Bartolini A Cavallaro A Palu G Ultrarapid detection of blaKPC(1)/(2)-(1)(2) from perirectal and nasal swabs by use of real-time PCR J Clin Microbiol 2012 50 1718 1720 10.1128/JCM.00195-12 22378915 PMC3347151

[B18] Richter SN Frasson I Franchin E Bergo C Lavezzo E Barzon L Cavallaro A Palu G KPC-mediated resistance in Klebsiella pneumoniae in two hospitals in Padua, Italy, June 2009-December 2011: massive spreading of a KPC-3-encoding plasmid and involvement of non-intensive care units Gut Pathog 2012 4 7 10.1186/1757-4749-4-7 22800501 PMC3411510

[B19] Birgy A Bidet P Genel N Doit C Decre D Arlet G Bingen E Phenotypic screening of carbapenemases and associated beta-lactamases in carbapenem-resistant Enterobacteriaceae J Clin Microbiol 2012 50 1295 1302 10.1128/JCM.06131-11 22259214 PMC3318498

[B20] van Dijk K Voets GM Scharringa J Voskuil S Fluit AC Rottier WC Leverstein-Van Hall MA Cohen Stuart JW A disc diffusion assay for detection of class A, B and OXA-48 carbapenemases in Enterobacteriaceae using phenyl boronic acid, dipicolinic acid and temocillin Clin Microbiol Infect 2014 20 345 349 10.1111/1469-0691.12322 23927659

[B21] Doyle D Peirano G Lascols C Lloyd T Church DL Pitout JD Laboratory detection of Enterobacteriaceae that produce carbapenemases J Clin Microbiol 2012 50 3877 3880 10.1128/JCM.02117-12 22993175 PMC3503014

[B22] Canton R Akova M Carmeli Y Giske CG Glupczynski Y Gniadkowski M Livermore DM Miriagou V Naas T Rossolini GM Samuelsen Ø Seifert H Woodford N Nordmann P European Network on Carbapenemases Rapid evolution and spread of carbapenemases among Enterobacteriaceae in Europe Clin Microbiol Infect 2012 18 413 431 10.1111/j.1469-0691.2012.03821.x 22507109

[B23] Potron A Poirel L Rondinaud E Nordmann P Intercontinental spread of OXA-48 beta-lactamase-producing Enterobacteriaceae over a 11-year period, 2001 to 2011 Surveill 2013 18 10.2807/1560-7917.es2013.18.31.20549 23929228

[B24] Jacoby GA AmpC beta-lactamases Clin Microbiol Rev s 2009 22 161 182 10.1128/CMR.00036-08 PMC2620637 19136439

[B25] Vading M Samuelsen O Haldorsen B Sundsfjord AS Giske CG Comparison of disk diffusion, Etest and VITEK2 for detection of carbapenemase-producing Klebsiella pneumoniae with the EUCAST and CLSI breakpoint systems Clin Microbiol Infect 2011 17 668 674 10.1111/j.1469-0691.2010.03299.x 20649801

[B26] Nordmann P Gniadkowski M Giske CG Poirel L Woodford N Miriagou V European Network on C Identification and screening of carbapenemase-producing Enterobacteriaceae Clin Microbiol Infect 2012 18 432 438 10.1111/j.1469-0691.2012.03815.x 22507110

[B27] Girlich D Poirel L Nordmann P Value of the modified Hodge test for detection of emerging carbapenemases in Enterobacteriaceae J Clin Microbiol 2012 50 477 479 10.1128/JCM.05247-11 22116154 PMC3264163

[B28] Liao IC Chen HM Wu JJ Tsai PF Wang LR Yan JJ Metallo-beta-lactamase-producing Enterobacteriaceae isolates at a Taiwanese hospital: lack of distinctive phenotypes for screening APMIS 2011 119 543 550 10.1111/j.1600-0463.2011.02772.x 21749455

[B29] Peter S Lacher A Marschal M Holzl F Buhl M Autenrieth I Kaase M Willmann M Evaluation of phenotypic detection methods for metallo-beta-lactamases (MBLs) in clinical isolates of Pseudomonas aeruginosa Eur J Clin Microbiol Infect Dis 2014 [Epub ahead of print] 10.1007/s10096-014-2059-1 24452967

[B30] Mammeri H Nordmann P Berkani A Eb F Contribution of extended-spectrum AmpC (ESAC) beta-lactamases to carbapenem resistance in Escherichia coli FEMS Microbiol Lett 2008 282 238 240 10.1111/j.1574-6968.2008.01126.x 18371063

[B31] Poirel L Bonnin RA Nordmann P Rapid identification of antibiotic-resistant bacteria: how could new diagnostic tests halt potential endemics? Expert Rev Mol Diagn 2013 13 409 411 10.1586/erm.13.30 23782246

[B32] Perez-Perez FJ Hanson ND Detection of plasmid-mediated AmpC beta-lactamase genes in clinical isolates by using multiplex PCR J Clin Microbiol 2002 40 2153 2162 10.1128/JCM.40.6.2153-2162.2002 12037080 PMC130804

[B33] Naas T Ergani A Carrer A Nordmann P Real-time PCR for detection of NDM-1 carbapenemase genes from spiked stool samples Antimicrob Agents Chemother 2011 55 4038 4043 10.1128/AAC.01734-10 21690281 PMC3165338

[B34] Tsakris A Poulou A Pournaras S Voulgari E Vrioni G Themeli-Digalaki K Petropoulou D Sofianou D A simple phenotypic method for the differentiation of metallo-beta-lactamases and class A KPC carbapenemases in Enterobacteriaceae clinical isolates J Antimicrob Chemother 2010 65 1664 1671 10.1093/jac/dkq210 20542902

